# Adherence to diet recommendations and risk of abdominal aortic aneurysm in the Malmö Diet and Cancer Study

**DOI:** 10.1038/s41598-018-20415-z

**Published:** 2018-01-31

**Authors:** Sara Nordkvist, Emily Sonestedt, Stefan Acosta

**Affiliations:** 1 Department of Clinical Sciences, Lund University, Malmö, Sweden; 2 Vascular Centre, Department of Cardiothoracic and Vascular Surgery, Skåne University Hospital, Malmö, Sweden

## Abstract

The research examining the association between quality of diet and abdominal aortic aneurysm (AAA) is scarce. The aim of the present study was to explore the association between diet quality and development of AAA for middle-aged individuals in the Malmö Diet and Cancer Study (MDCS), a prospective cohort study with baseline data collection carried out between 1991 and 1996. At baseline, the study participants who were eligible for this study (n = 26133) documented their dietary habits in a food diary and questionnaire. Incident AAA cases during an average of 20.7 years of follow-up were identified by using registers. A diet quality index consisting of six components, saturated fat, polyunsaturated fat, fibre, sucrose, fruits and vegetables and fish and shellfish, was used to assess the diet quality. After adjusting for potential confounders, the diet quality index was not associated with incident AAA. However, a tendency of decreased risk was observed among individuals adhering to recommendations for fruit and vegetables compared with non-adherence. When comparing the risk of more extreme intake groups, high intakes of both fruits and vegetables were associated with decreased risk.

## Introduction

Abdominal Aortic Aneurysm (AAA) is most commonly defined as an aortic diameter of 3.0 centimetres or more^1^. The pathogenesis of AAA is not clearly understood, and an AAA usually grows slowly and remains asymptomatic until rupture occurs. The overall mortality of ruptured AAA is still very high, at approximately 81%^2^.

Smoking is a strong and established risk factor of AAA, and aneurysm growth rate in current smokers was 0.35 mm/year faster than in ex- or never smokers^3^. Therefore, smoking cessation is the most established and effective method for diminishing growth rate^4^. Male sex is another risk factor for AAA and it was assessed that AAA is four to six times more common in men compared to women^3,5^. Most clinical AAA cases are aged above 65^6^. Because of these risk factors, United Kingdom and Sweden have implemented screening with ultrasound for men aged 65 and beyond^7^.

Maintaining a high-quality diet has been shown to reduce the risk of many conditions, such as cardiovascular disease and diabetes^8,9^. However, studies examining the association between dietary habits and risk of developing AAA is very limited. A retrospective cross-sectional cohort study conducted in the United States, comprising over 3 million individuals, showed that a high consumption of fruits, vegetables and nuts decreased the risk of AAA^10^. In a prospective cohort study conducted in Sweden it was reported that a high intake of fruits, but not vegetables, reduces the risk of AAA^11^. The association between diet quality and incident cardiovascular disease in the Malmö Diet and Cancer Study (MDCS) has previously been explored^12^, but no research has focused on diet quality and incident AAA.

The aim of the present population-based prospective study on middle-aged individuals in the MDCS was to explore the association between diet quality, specific dietary components and development of AAA.

## Method

### Study population and data collection

**Figure 1:**
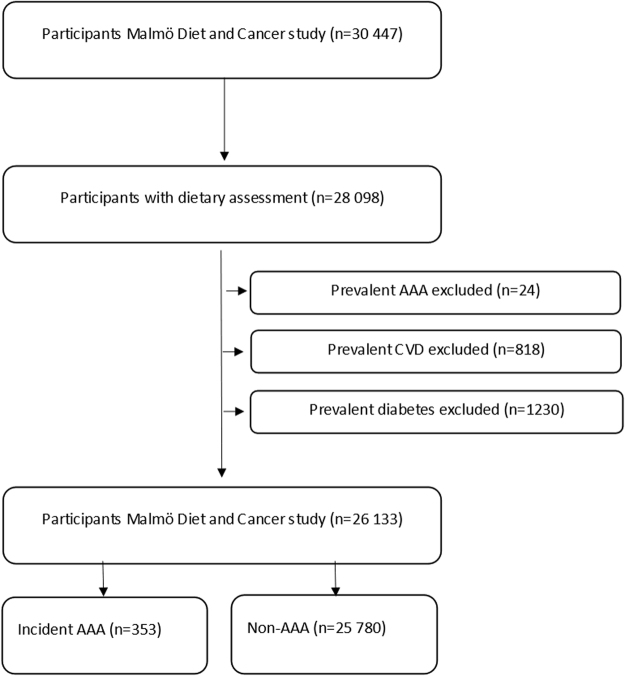
Descriptive flow diagram of study participants, dietary data and exclusions. AAA indicates abdominal aortic aneurysm and CVD indicates cardiovascular disease. Some individuals had multiple exclusion criteria.

The Malmö Diet and Cancer Study (MDCS) was a prospective cohort study and baseline sampling was carried out between 1991 and 1996. Men born between 1923 and 1945 and women born between 1923 and 1950 were eligible to participate in the study. The participants had to be proficient in Swedish and reside in Malmö, Sweden. In total 30446 participants were recruited, out of which 28098 participated in diet assessment, anthropometric measurements and answered a comprehensive questionnaire. More detailed descriptions of the recruitment and data collection process can be found elsewhere^13,14^. Participants with prevalent AAA, cardiovascular disease (prior myocardial infarction and stroke) or diabetes mellitus (higher likelihood to change dietary habits) were excluded from the study population. This resulted in a total study population of 26133 (Fig. 1). All participants gave informed consent and the study had ethical clearance from the Regional Ethical Review Board in Lund, Sweden (Dnr §LU5190). The study protocol conformed to the ethical guidelines of the 1975 Declaration of Helsinki.

### Diet assessment method and construction of the diet quality index

Information on dietary habits was collected through a seven-day food diary where the participant recorded their food intake at lunch and dinner meals and intake of cold beverages, together with a 168-item food frequency questionnaire where frequency and portion-size of foods regularly consumed during the past year were documented^15^. The food diary was complemented with a one-hour interview which collected detailed data on for instance serving sizes and cooking practises^16^. The methodology used when developing the diet quality index have been described in detail previously^16^. Briefly, candidate components had to be available within the MDCS cohort. Trans fatty acids and salt intake had to be excluded, since information on these dietary factors either was lacking or had poor validity. Second, dietary components considered most important in assuring overall diet quality (i.e. food groups and macronutrients) in relation to chronic disease were primarily considered. Third, inter-correlation between components were investigated to assess the mutual independence between components. Considering these three aspects, six dietary components were selected: Saturated fat, polyunsaturated fat, fish and shellfish, dietary fibre, fruit and vegetables, and sucrose. Individuals who reached the recommendations for each of the dietary component outlined in the Swedish nutrition recommendations and the Swedish dietary guidelines, were given one point. For saturated fat, an adjusted cut-off point was used, since only 2% of the study population reached the recommendation levels. The cut-offs for reaching the recommendations were therefore: polyunsaturated fat 5–10 E%, fish and shellfish ≥300 g/week, sucrose ≤10 E%, dietary fibre ≥2.4 g/MJ, saturated fat ≤14 E%, fruit and vegetables (fruit juices excluded) ≥400 g/d. A maximum score of six was attainable in the index, given that the individual adheres to the recommendations for each dietary component. The quality index was also categorised as low (0–1 points), medium (2–4 points) and high (5–6 points). Previous research has found that maintaining a high score on the diet quality index leads to decreased mortality^17^ and to lower rates of systemic inflammations, which can be directly associated with incidence of cancer and cardiovascular disease^18^.

### Endpoint ascertainment

The personal number of the participants in the MDCS were linked with registries logging first diagnosis of AAA, ruptured AAA or surgical procedure for AAA. The included registries are the Cause of Death Register and the Inpatient and Outpatient Register. The cause of Death Registers included all registered deaths in Sweden as well as the cause of death as noted in the death certificate. The Inpatient and Outpatient Register contained records of all hospitalizations in Sweden as well as diagnostic and procedural codes. A revised version of the International Classification of Disease (ICD), version 8, 9 and 10, was used for both registers. The surgical codes are based on a Swedish classification system.

### Other variables

Age and sex was determined by procuring the civic registration numbers of each participant. The weight and height of all participants was registered by nurses and body mass index (BMI) was calculated using the formula kg divided by m^2^ expressed in kg per m^2^. Hypertension was defined as systolic blood pressure ≥130 mm Hg or diastolic blood pressure ≥85 mmHg DBP or antihypertensive drug treatment. Lifestyle variables were assessed through a self-administered questionnaire. Smoking habits were categorised as never, former and current smoker. Time since smoking cessation was stratified in tertiles and used to define a smoking score. A smoking score ranging from 0 to 4 was established: 0 = never smokers; 1 = no smoking since 25–51 years; 2 = no smoking since 12–24 years; 3 = no smoking since 1–11 years; 4 = currently smoking. In a subgroup of individuals in the present study, this smoking score was highly associated with pack-years smoked^19^. The participants were divided into five categories based on the highest education level attained, i.e. less than nine years, elementary school (9–10 y), upper secondary school (11–13 y), university without a degree and university degree. Leisure-time physical activity level was expressed as Metabolic equivalent of task (MET) hours per week based on intensity level and time spent at 17 different activities and was divided into five groups. Alcohol habits was divided into six groups. Those who had not consumed alcohol in the last year were categorised as zero-consumers and the rest were divided into gender-specific quintiles according to their intake as reported in the seven-day food diary. Total energy intake was kcal/day from diet and supplement, including alcohol and fibre. Season refers to time of year that the data collection took place and dietary assessment method refers to the change in coding practice after 1 September 1994^15^. Non-adequate reporters of energy were identified by comparing their reported energy intake with their total energy expenditure (i.e., estimated from their calculated basal metabolic rate and self-reports of leisure-time physical activity, work activity, household work, and sleep hours). Individuals with reported energy intake above or below the 95% confidence interval (CI) for total energy expenditure were categorized as “misreporters”. Individuals answering yes to the questionnaire item “Have you substantially changed your dietary habits in the past?” were classified as “dietary changers”^20^.

### Statistical analysis

The baseline characteristics for sex, age, BMI, diet and lifestyle variables were presented as median and interquartile range (IQR) for the continuous variables and as total count and percentage for the categorical variables. For these variables, we also calculated hazard ratio (HR) by using the Cox proportional hazards regression model with years of follow-up as the time scale. For age, BMI and dietary variables, HR per one standard deviation (SD) was calculated. HR for diet variables were adjusted for age, sex, diet assessment method, season and total energy intake and HR for lifestyle factors, hypertension and medication were adjusted for age and sex. The HR for diet quality index categories (low, medium and high) and according to non-adherence and adherence to each dietary recommendation was calculated and adjusted for sex, total energy intake, diet assessment method and season in the basic model, and alcohol consumption, physical activity, smoking status, education level and BMI were added in the extended multivariable model. The mutually adjusted model with the six dietary components had, in addition to the adjusted variables in the multivariable model, been mutually adjusted for the other five diet quality index components. The statistical analyses were carried out in IBM SPSS, version 22 (SPSS Inc, Chicago, IL). The chosen level of statistical significance was 0.05.

## Results

### Demographic baseline characteristics

In the study population of 26133, 353 (1.4%) had been diagnosed with AAA, during a median follow-up time of 20.7 years (IQR 19.7–21.4). Baseline characteristics of diet variables and life-style factors for AAA individuals and non-AAA individuals are outlined in Table 1. Individuals that developed AAA during follow-up were older, were more often males and had higher BMI than non-AAA individuals. Current smoking at baseline was strongly associated with higher incidence of AAA (HR 9.26; 95% CI 6.81–13.57) compared with never smoking. Individuals with incident AAA were less educated and had a lower degree of leisure-time physical activity than non-AAA individuals (Table 1). Intakes of fibre and fruit and vegetables were lower, and intake of polyunsaturated fat higher among individuals that were diagnosed with AAA compared to the rest of the study population.

**Table 1:** Baseline characteristics of incident AAA cases (n = 353) and non-AAA cases (n = 25780) in the Malmö Diet and Cancer cohort.

	AAA cases	Non-cases	*HR (95% CI)*
Males (%)	260 (73.7)	9616 (37)	4.68 (3.69–5.93)
Age (years)	62.14 (9.45)	57.22 (12.91)	2.20 (1.94–2.50)
BMI (kg/m^2^)	25.56 (4.59)	25.15 (4.83)	1.12 (1.00–1.25)
Hypertension	318 (90.1)	19646 (76.2)	1.80 (1.27–2.57)
Saturated fat (E%)	15.75 (5.14)	15.82 (4.73)	0.96 (0.87–1.07)
Polyunsaturated fat (E%)	6.07 (2.15)	5.73 (1.97)	1.11 (1.00–1.22)
Fish and shellfish (g/week)	290.81 (290.87)	276.67 (287.76)	1.03 (0.93–1.14)
Fibre (g/MJ)	1.87 (0.84)	2.13 (0.78)	0.86 (0.76–1.00)
Fruits and vegetables (g/day)	274.25 (197.30)	347.83 (228.72)	0.77 (0.67–0.88)
Sucrose (E%)	7.76 (5.25)	8.12 (4.21)	1.01 (0.91–1.11)
Alcohol consumption			
Zero-consumption	23 (6.5)	1574 (6.1)	1.00
Quintile 1	70 (19.8)	4728 (18.3)	0.83 (0.51–1.32)
Quintile 2	65 (18.4)	4772 (18.5)	0.71 (0.44–1.15)
Quintile 3	68 (19.3)	4889 (19)	0.75 (0.47–1.20)
Quintile 4	62 (17.6)	4892 (19)	0.71 (0.44–1.16)
Quintile 5	65 (18.4)	4925 (19.1)	0.86 (0.54–1.40)
Smoking status			
Never	39 (11)	10037 (38.9)	1.00
Current	214 (60.6)	7223 (28)	9.26 (6.81–13.57)
Former	100 (28.3)	8510 (33)	2.46 (1.70–3.58)
Smoking score (0–4)	3.03 (1.42)	1.77 (1.68)	1.66 (1.54–1.79)
Educational level			
Less than 9 years	196 (55.7)	10537 (40.9)	1.00
Elementary school (9–10 y)	68 (19.3)	6836 (26.5)	0.72 (0.54–0.94)
Upper secondary school (11–13 y)	25 (7.1)	2303 (8.9)	0.57 (0.38–0.87)
University without a degree	27 (7.7)	2274 (8.8)	0.72 (0.48–1.08)
University degree	35 (10.2)	3772 (14.6)	0.67 (0.47–0.95)
Leisure-time physical activity			
0–7.5 MET-h/week	51 (14.6)	2394 (9.4)	1.00
7.5–15 MET-h/week	60 (17.1)	3819 (14.9)	0.72 (0.35–1.04)
15–25 MET-h/week	66 (18.9)	5896 (23)	0.50 (0.35–0.72)
25–50 MET-h/week	120 (34.3)	9397 (36.7)	0.54 (0.39–0.74)
>50 MET-h/week	53 (15.1)	4098 (16)	0.46 (0.31–0.68)
Use of lipid lowering drugs	26 (7.4)	534 (2.1)	2.52 (1.68–3.76)
Use of statins	17 (4.8)	344 (1.3)	2.42 (1.48–3.94)
Use of acetyl salicylic acid for cardiovascular disease	14 (4.0)	249 (1.0)	2.91 (1.70–5.00)

Data are median (IQR), n (%) or mean (SD).

HR for diet variables adjusted for age, sex, diet assessment method, season, energy intake.

HR per 1 SD increment was calculated for age, BMI and dietary intakes.

HR for lifestyle factors, hypertension and medications adjusted for age and sex.

### Diet quality index and AAA risk

In the basic model, the diet quality index was associated with a decreased AAA risk (p = 0.03). The association was attenuated in the full multivariable model adjusting for several potential confounders (p = 0.68) (Table 2) and remained the same in a sensitivity analysis after including hypertension, acetyl salicylic acid (ASA) and statins into the full multivariable model (p = 0.70). The risk estimates associated with a 1-point increase in the dietary score was for the basic model 0.89 (95% CI 0.82–0.96) and multi-variable model 0.98 (95% CI 0.91–1.07).

**Table 2:** HR and 95% CI for incident AAA by categories of diet quality index (low, medium, high) among participants in the Malmö Diet and Cancer cohort.

	Low (0–1 points)	Medium (2–4 points)	High (5–6 points)	*P* trend
Cases/Non-cases	61/4045	257/18425	35/3310	
Basic model^a^	1.00	0.82 (0.62–1.09)	0.62 (0.41–1.95)	0.03
Multivariable model^b^	1.00	0.96 (0.72–1.27)	0.96 (0.63–1.47)	0.68

^a^Adjustments for age, sex, total energy intake, diet assessment method, and season.

^b^Adjustments for age, sex, total energy intake, diet assessment method, season, alcohol consumption, physical activity, smoking, education, and BMI.

### Adherence to components of diet index quality recommendations and AAA risk

In the basic model, individuals that adhered to the recommended intake of fibre (HR 0.68; 95% CI 0.53–0.88) and fruits and vegetables (HR 0.59; 95% CI 0.46–0.76) had significantly lower risk of AAA (Table 3). In the multivariable and mutually adjusted multivariable model, a trend remained for adherence to fruits and vegetables and a lower AAA risk (p = 0.07). In a sensitivity analysis, including hypertension, ASA and statins in the mutually adjusted multivariable model, the trend remained (p = 0.07). To compare more extreme intakes, fruits and vegetables were separately divided into four groups (<100 g/day, 100–200 g/day, 200–300 g/day, >300 g/day). Both high fruit and vegetable intake was associated with a lower AAA risk in the multi-variable model (p = 0.017 and 0.006, respectively) with the following HR and 95% CI for fruit intakes: 1.00 (ref), 0.76 (0.59–0.98), 0.63 (0.46–0.88), 0.67 (0.46–0.98) and for vegetable intakes: 1.00 (ref), 0.81 (0.63–1.05), 0.74 (0.53–1.03), 0.60 (0.37–0.98). The associations were virtually unchanged when adjusted for vegetable or fruit intake, respectively.

**Table 3:** HR and 95% CI for incident AAA by adherence to diet quality index components among participants in the Malmö Diet and Cancer cohort.

Dietary components	Non-adherence	Adherence
**Saturated fat**	**≥14 E%**	**≤14 E%**
Cases/Non-cases	250/18401	103/7379
Basic model^a^	1.00	0.96 (0.76–1.22)
Multivariable model^b^	1.00	1.16 (0.92–1.48)
Mutually adjusted multivariable model^c^	1.00	1.24 (0.95–1.61)
**Polyunsaturated fat**	**<5 E% or >10 E%)**	**5–10 E%**
Cases/Non-cases	92/8106	261/17674
Basic model^a^	1.00	1.13 (0.89–1.43)
Multivariable model^b^	1.00	1.10 (0.86–1.40)
Mutually adjusted multivariable model^c^	1.00	1.09 (0.86–1.40)
**Fish and shellfish**	**≤300 g/week**	**≥300 g/week**
Cases/Non-cases	187/14095	166/11685
Basic model^a^	1.00	0.89 (0.72–1.11)
Multivariable model^b^	1.00	0.93 (0.75–1.15)
Mutually adjusted multivariable model^c^	1.00	0.93 (0.75–1.15)
**Fibre**	**≤2.4 g/MJ**	**≥2.4 g/MJ**
Cases/Non-cases	271/17266	82/8514
Basic model^a^	1.00	0.68 (0.53–0.88)
Multivariable model^b^	1.00	0.94 (0.73–1.22)
Mutually adjusted multivariable model^c^	1.00	0.97 (0.71–1.32)
**Fruits and vegetables**	**≤400 g/day**	**≥400 g/day**
Cases/Non-cases	269/15880	84/9900
Basic model^a^	1.00	0.59 (0.46–0.76)
Multivariable model^b^	1.00	0.79 (0.61–1.02)
Mutually adjusted multivariable model^c^	1.00	0.78 (0.59–1.03)
**Sucrose**	**≥10 E%**	**≤10 E %**
Cases/Non-cases	102/7415	251/18365
Basic model^a^	1.00	0.89 (0.70–1.12)
Multivariable model^b^	1.00	0.97 (0.77–1.23)
Mutually adjusted multivariable model^c^	1.00	0.99 (0.78–1.27)

^a^Adjusted for age, sex, total energy intake, diet assessment method, and season.

^b^Adjusted for age, sex, total energy intake, diet assessment method, season, alcohol consumption, physical activity, smoking, education and BMI.

^c^Adjusted for age, sex, total energy intake, diet assessment method, season, alcohol consumption, physical activity, smoking, education, BMI and mutual adjustment for the six diet quality index components.

### Sensitivity analysis

A sensitivity analysis was performed after removing misreporters (n = 4799) and dietary changers (n = 5623). The association between diet quality index, the six dietary components and incident AAA was virtually unchanged (supplementary Tables 4 and 5).

## Discussion

After adjusting for several confounders, Individuals with the highest diet quality score did not have a decreased risk of incident AAA. A tendency of decreased risk was observed among individuals adhering to recommendations for fruit and vegetables compared with non-adherence. When comparing the risk of more extreme intake groups, the highest intake group of fruits (<300 g/day) was associated with 33% decreased risk. The corresponding group for vegetable intake was associated with 40% decreased risk.

The finding regarding fruit consumption is consistent with the findings from another Swedish cohort study, consisting of 80 446 individuals out of which 1086 were incident AAA cases (cumulative AAA incidence 1.3%)^11^. That study reported that when comparing the group with the highest intake of fruits (>2 servings/day) with the lowest intake group (<0.7 servings/day), the highest group had a 25% decreased risk of incident AAA. A finding consistent with the current study. However, in contrast to that report, which did not identify an association between vegetable consumption and AAA risk^11^, the present study found that vegetable consumption was associated with lower AAA risk. A potential explanation to the deviations in results can be that the current study has a different diet assessment method compared to the other Swedish cohort study. In addition, it is possible that high consumers of fruit and vegetables has other way of life style than low consumers, that not has been taken into account in the multi-variable analysis, that may explain the protective effect towards AAA development.

The role of antioxidants and their ability to reduced oxidative stress in the aortic wall is one plausible theory that has been suggested as a protective factor of fruit and vegetables in AAA pathogenesis, but there is no hard evidence as there is a paucity of conducted experimental studies on this topic^21^. In this context, it should also be kept in mind that fruit and vegetables contains numerous types and varying concentrations of antioxidants and other bioactive compounds, for instance flavonoids such as procyanidins^22,23^ and quercetin^24^.

A diet with low quality was in the basic statistical model associated with increased AAA risk, which is in line with the large retrospective cross-sectional cohort study reported by Kent *et al*.^10^. The study conducted by Kent *et al*. investigated AAA risk for individuals, in total approximately 3 million, who had previously filled in a medical and lifestyle questionnaire and were screened for AAA. The study did not have an explicit focus on diet but analysed a plethora of potential risk factors, including for instance excess weight, ethnicity and family history. It was concluded that smoking cessation and a healthy lifestyle, including consumption of nuts, fruits and vegetables decreased AAA risk. The present study had detailed information on a large number of confounders that were included in the adjusted models. The association between a low-quality diet and AAA was attenuated in the multivariable model, which indicated that other lifestyle factors impact AAA risk. This is contemporaneous with the study by Kent *et al*.

In the basic model, fibre intake lowered risk of AAA, but the association disappeared when further adjustments were made. A positive strong correlation between fruit and vegetable and fibre intake in the MDCS has been previously identified^16^, and the association between fibre and AAA risk was clearly attenuated after adjusting for the other diet components, including fruit and vegetables. Further analysis of other food sources of fibre intake, including fibre rich cereal products^25^ and incident AAA in MDCS cohort is warranted. It is noteworthy that intake of dietary fibre, fruit and vegetables is higher for women than for men^26^ possibly contributing to the, in general, lower incidence and later development of AAA in women compared to men^7^.

The present study design with AAA ascertainment by hospital registers, reflects identification of large-sized AAA and AAA risk in its later stages. Therefore, one of the limitations of the study is the absence of ultrasound measurement of AAA diameter at baseline and we can therefore not exclude the possibility of erroneously including small AAA at study entry. To minimize this bias, however, patients with cardiovascular disease, a co-morbidity highly correlated with concomitant AAA^27–29^ at baseline were excluded. Moreover, the non-randomized, observational study design does not allow causal interpretations, which is another limitation to the present study.

The cohort was designed to study dietary habits, specifically fibre and fat quality, in a middle-aged and older population. However, self-reported dietary intake data are likely to be associated with some degree of misclassification and may have changed during follow up. Individuals that might have changed the dietary habits, such as patients with known diabetes mellitus, myocardial infarction or stroke, were excluded at baseline. Of note, lower extremity artery disease, a less common atherosclerotic cardiovascular morbidity, than both coronary artery disease and stroke^30^, was not excluded. On the other hand, individuals with lower extremity artery disease are less exposed to and aware of benefits of dietary interventions compared to individuals with coronary artery disease and stroke^31^. Hence, it is likely that there will be few dietary changers among the low number of individuals with lower extremity artery disease without other atherosclerotic manifestations in the MDCS cohort. In addition, a sensitivity analysis was performed, excluding misreporters and dietary changers. This analysis showed that when these individuals were excluded, the associations between the diet quality index, dietary components and incident AAA were unchanged, which confirms the findings from the whole population on diet and AAA risk. Excluding misreporters and dietary changers has been shown to result in a stable population on food habits throughout the follow-up period^20^.

One might argue that a supplementary evaluation of other established healthy indices such as the Mediterranean Diet score^32^ or Dietary Approach to Stop Hypertension (DASH)^33^ in relation to development of AAA would have strengthened the present study. The present diet quality index was developed to particularly assess adherence to the Swedish nutrition recommendations and the Swedish dietary guidelines^16^. This diet quality index was found to be valid and showed that a high diet quality was associated with a decreased risk of cardiovascular events^12^ in the MDCS. As a next step, it was considered warranted to evaluate the utility of this, in comparison to the above-mentioned indices, simple tool for assessment of incident AAA, a disease related to the same risk factors as those with cardiovascular events^12^. It was not possible to show that high-quality diet was associated with decreased AAA risk, which probably may be due to a too low number of individuals with AAA during follow up, leading to a type 2 statistical error. Perhaps a future analysis with a higher cumulative incidence of AAA would yield another result.

Besides baseline measurement, repeated measurements of life style modifiable risk factors for AAA would have been valuable, since for instance smoking prevalence has decreased^7^ and intakes of fruit, berries and vegetables may have increased in recent times^34^. The cumulative AAA incidence of 1.4% in the present cohort study is relatively high in view of the predominance of female individuals. This can be compared with the current AAA prevalence of 1.5% among 65-year old ultrasound screened Swedish men^35^. The large sample size, the long follow up time and the high relative validity of the dietary intake assessment^34,36^ are other factors that strengthen the present study. Finally, the individuals were middle-aged or older at baseline, which means that the extension of follow-up period ensured evaluation of most males beyond 80 years, an age where AAA incidence for men peaks^37^.

In conclusion, while individuals with the highest diet quality score did not reduce their AAA risk, high intakes of vegetables and fruits decreased the risk of incident AAA in the present study.

## Electronic supplementary material

Supplementary table 4 and table 5
