# Author Correction: Adherence to diet recommendations and risk of abdominal aortic aneurysm in the Malmö Diet and Cancer Study

**DOI:** 10.1038/s41598-018-25616-0

**Published:** 2018-05-15

**Authors:** Sara Nordkvist, Emily Sonestedt, Stefan Acosta

**Affiliations:** 1 Department of Clinical Sciences, Lund University, Malmö, Sweden; 2 Vascular Centre, Department of Cardiothoracic and Vascular Surgery, Skåne University Hospital, Malmö, Sweden

Correction to: *Scientific Reports* 10.1038/s41598-018-20415-z, published online 31 January 2018

This Article contains typographical errors.

In the Discussion section,

“When comparing the risk of more extreme intake groups, the highest intake group of fruits (<300 g/day) was associated with 33% decreased risk.”

should read:

“When comparing the risk of more extreme intake groups, the highest intake group of fruits (>300 g/day) was associated with 33% decreased risk.”

