# Supplementary material for: Adherence to diet recommendations and risk of abdominal aortic aneurysm in the Malmö Diet and Cancer Study
Source: Sci Rep. 2018 Jan 31;8:2017. doi: 10.1038/s41598-018-20415-z (PMC5792541; doi:10.1038/s41598-018-20415-z)
Supplement: Supplementary file 1 — Supplementary table 4 and table 5 [file 41598_2018_20415_MOESM1_ESM.doc]

**Adherence to diet recommendations and risk of abdominal aortic aneurysm in the Malmö Diet and Cancer Study**

Sara Nordkvist^1^, Emily Sonestedt^1^, Stefan Acosta^1,2^.

Department of Clinical Sciences, Malmö, Lund University^1^, Sweden. Vascular Centre^2^, Department of Cardiothoracic and Vascular Surgery, Skåne University Hospital, Sweden.

Corresponding author:

Sara Nordkvist

Department of Clinical Sciences, Malmö, Lund University, Sweden.

Email: sara.nordkvist@med.lu.se

**Table 4:** HR and 95% CI for incident AAA by categories of diet quality index (low, medium, high) among participants in the Malmö Diet and Cancer cohort, dietary changers and misreporters were excluded

^a^Adjustments for age, sex, total energy intake, diet assessment method, and season

|  | **Low** | **Medium** | **High** | ***P* trend** |
| --- | --- | --- | --- | --- |
| Cases/Non-cases | 42/2847 | 175/12152 | 17/1692 |  |
| Basic model^a^ | 1.00 | 0.86 (0.61-1.2) | 0.58 (0.33-1.02) | 0.01 |
| Multivariable model ^b^ | 1.00 | 0.98 (0.70-1.39) | 0.84 (0.47-1.49) | 0.40 |

^b^Adjustments for age, sex, total energy intake, diet assessment method, season, alcohol consumption, physical activity, smoking, education, and BMI

**Table 5:** HR and 95% CI for incident AAA by adherence to diet quality index components among participants in the Malmö Diet and Cancer cohort, dietary changers and misreporters were excluded

| **Dietary components** | **Non-adherence** | **Adherence** |
| --- | --- | --- |
| **Saturated fat** | **≥ 14 E %** | **≤ 14 E %** |
| Cases/Non-cases | 183/12883 | 51/3808 |
| Basic model^a^ | 1.00 | 0.82 (0.60-1.13) |
| Multivariable model^b^ | 1.00 | 0.96 (0.70-1.32) |
| Mutually adjusted multivariable model^c^ | 1.00 | 1.02 (0.73-1.44) |
| **Polyunsaturated fat** | **<5 E% or >10 E %)** | **5-10 E%** |
| Cases/Non-cases | 54/4959 | 180/11732 |
| Basic model^a^ | 1.00 | 1.22 (0.90-1.66) |
| Multivariable model^b^ | 1.00 | 1.18 (0.87-1.61) |
| Mutually adjusted multivariable model^c^ | 1.00 | 1.18 (0.86-1.61) |
| **Fish and shellfish** | **≤300g/week** | **≥300g/week** |
| Cases/Non-cases | 129/8945 | 105/7746 |
| Basic model^a^ | 1.00 | 0.80 (0.61-1.02) |
| Multivariable model^b^ | 1.00 | 0.82 (0.63-1.07) |
| Mutually adjusted multivariable model^c^ | 1.00 | 0.82 (0.63-1.08) |
| **Fibre** | **≤2.4 g/MJ** | **≥2.4 g/MJ** |
| Cases/Non-cases | 191/12252 | 43/4439 |
| Basic model^a^ | 1.00 | 0.66 (0.47-0.92) |
| Multivariable model^b^ | 1.00 | 0.89 (0.63-1.25) |
| Mutually adjusted multivariable model^c^ | 1.00 | 0.98 (0.66-1.45) |
| **Fruits and vegetables** | **≤400g/day** | **≥400g/day** |
| Cases/Non-cases | 183/10761 | 51/5930 |
| Basic model^a^ | 1.00 | 0.59 (0.43-0.82) |
| Multivariable model^b^ | 1.00 | 0.77 (0.56-1.07) |
| Mutually adjusted multivariable model^c^ | 1.00 | 0.82 (0.63-1.08) |
| **Sucrose** | **≥10 E%** | **≤10 E %** |
| Cases/Non-cases | 64/4917 | 170/11774 |
| Basic model^a^ | 1.00 | 0.99 (0.74-1.32) |
| Multivariable model^b^ | 1.00 | 1.03 (0.80-1.45) |
| Mutually adjusted multivariable model^c^ | 1.00 | 1.01 (0.80-1.47) |

^a^Adjusted for age, sex, total energy intake, diet assessment method, and season

^b^Adjusted for age, sex, total energy intake, diet assessment method, season, alcohol consumption, physical activity, smoking, education and BMI

^c^Adjusted for age, sex, total energy intake, diet assessment method, season, alcohol consumption, physical activity, smoking, education, BMI and mutual adjustment for the six diet quality index components
